# How Primary Care Clinicians Process Patient Death: Logistics, Emotions, and Opportunities for Structural Support

**DOI:** 10.1007/s11606-024-08702-0

**Published:** 2024-03-08

**Authors:** Jessica Alcalay Erickson, Bridget C. O’Brien, Sarah Nouri

**Affiliations:** 1 Department of Medicine, University of California San Francisco, San Francisco, CA USA; 2 Center for Faculty Educators, University of California San Francisco, San Francisco, CA USA; 3 Division of Palliative Medicine, Department of Medicine, University of California San Francisco, San Francisco, CA USA

**Keywords:** primary care, patient death, emotional processing, logistics, support

## Abstract

**Background:**

Navigating the logistics and emotional processing of a patient’s death is an inevitable part of many physicians’ roles. While research has primarily examined how inpatient clinicians cope with patient loss, little work has explored how primary care clinicians (PCCs) handle patient death in the outpatient setting, and what support resources could help PCCs process loss.

**Objective:**

To explore PCCs’ experiences with the logistics and emotional processing of patient deaths and suggestions for supportive resources.

**Design:**

Qualitative study using semi-structured interviews conducted between March and May 2023.

**Participants:**

Recruitment emails were sent to 136 PCCs (physicians and nurse practitioners) at three San Francisco academic primary care clinics. Twelve clinicians participated in the study.

**Approach:**

This study used a template analysis approach. Interview transcripts were analyzed in an iterative fashion to identify themes for how PCCs navigate patient death.

**Results:**

Participants (*n*=12) described outpatient death notification as inconsistent, delayed, and rife with uncertainty regarding subsequent actions. They felt various emotions, notably sadness and guilt, especially with deaths of young, vulnerable patients or those from preventable illnesses. Participants identified strategies for emotional processing and recommended improvements including clear procedural guidance, peer debriefings, and formal acknowledgements of deceased patients.

**Conclusions:**

Interviewing PCCs about their experiences following a patient death revealed key themes in logistical and emotional processing, and clinic resource recommendations to better support PCCs. Given the distinct characteristics of primary care—such as enduring patient relationships, greater isolation in ambulatory settings compared to inpatient environments, and rising burnout rates—enhancing guidance and support for PCCs is crucial to mitigate administrative burdens and grief after patient loss.

**Supplementary Information:**

The online version contains supplementary material available at 10.1007/s11606-024-08702-0.

## INTRODUCTION

Navigating the logistics and emotional processing of a patient’s death is an inevitable, and often challenging, part of many physicians’ jobs. ^1–4^ In this context, “logistics” encompasses the range of professional tasks, duties, and responsibilities clinicians undertake in response to a patient’s death, including administrative processes, care coordination, and family communication. While the prevention, postponement, preparation for, and support of patients and their loved ones during death are often paramount in a physician’s day-to-day work, the aftereffects of a patient’s death on clinicians are less studied and understood. ^3,5–7^

Of the limited work examining how clinicians navigate patient death, most has focused on inpatient practitioners such as hospitalists or ICU workers, ^3,8^ oncology and palliative care physicians ,^9,10^ and learners. ^11–14^ A few interventions supporting inpatient practitioners and learners’ processing of patient death such as “death rounds” ^8,11,12^, “death cafés”, ^15^ and real-time attending-led death debrief sessions ^1^ have been evaluated. Comparably few contemporary studies examine how primary care clinicians (PCCs) navigate patient deaths in outpatient settings, with most existing research being over 15 years old. ^7,16,17^

Although PCCs may not always witness their patient’s death firsthand in the ICU or hospice facility, they often have longitudinal, meaningful relationships with patients and family members spanning decades and generations. The loss of a long-time primary care patient can therefore cause significant distress. ^2,7^ Processing a patient’s death has an emotional impact on physicians, ^3,18^ and if unaddressed, may lead to physician burnout. ^19,20^

The experience of patient loss differs in an inpatient versus outpatient setting. In hospitals, clinicians are often present when patients die, in reach of team members who cared for the patient, and can provide in-person condolences to family members. In primary care, clinicians may hear of a patient’s death weeks to months later depending on where and how the patient died, ^21^ and rarely have opportunities to immediately process the loss with other practitioners who knew the patient. Immediate steps following a patient’s death may also differ for inpatient and outpatient clinicians. While clear logistical steps exist for hospital deaths such as death exam and documentation, notification of family, donor network, and occasionally medical examiner, little is known about clinician expectations following ambulatory patient deaths.

Our study aims to understand how PCCs navigate patient death in the outpatient setting and to identify resources to support them in this process. Our specific research questions are:

1. How do PCCs experience patient deaths from a logistical and emotional perspective?
2. What resources and supports could benefit PCCs when faced with patient deaths?

## METHODS

### Study Design

We conducted a cross-sectional qualitative study using an interpretivist approach. ^22^ This approach recognizes reality as a construction of social interactions rather than as a fixed truth. We used template analysis ^23^ to identify and organize hierarchical themes. The University of California San Francisco (UCSF) Institutional Review Board deemed the study exempt from review.

### Participants and Setting

We conducted the study at three UCSF-affiliated primary care clinics in San Francisco. With permission from medical directors, we sent recruitment emails to three clinic listservs (*n*=136 physicians and nurse practitioners or “PCCs”). PCCs with their own panels and who had experienced at least one ambulatory patient death were invited to participate. We interviewed all PCCs who volunteered.

### Data Collection

The semi-structured interview guide (Appendix) was developed by a qualitative research expert (BOB), a palliative care physician (SN), and a primary care physician (JAE) at UCSF. We used themes identified from the limited studies completed on this topic ^3,4,7,10,16,17,24^ to guide development of our interview questions. The interviews consisted of 15 questions divided into 4 sections: (1) Logistical processing of patient death, (2) Emotional processing of patient death, (3) Suggestions for clinical resources to support PCCs process patient death, and (4) Demographics. JAE piloted the interview guide with a PCC at UCSF and revised the guide for clarity and flow. Consent was verbally obtained from study participants prior to interview initiation. The first author (JAE) conducted all interviews on Zoom between March and May 2023. Interviews were transcribed using AI technology (Otter.AI), then de-identified and reviewed for accuracy by JAE. All participants received a $10 gift card.

### Data Analysis

We used template analysis to code interview transcripts and develop themes. We used the interview guide sections as an initial coding framework (logistics, emotions, and suggestions for improvement) and then inductively generated specific, sub-codes within these broad code categories based on our data (for example, “inconsistent notifications of patient death” as a sub-code under “logistics following patient death”). All three researchers independently coded three interview transcripts and subsequently met to discuss discrepancies and potential additions to the coding template. JAE refined the coding template based on this discussion. JAE entered the final coding template into Dedoose analytic software v.9.0.107 and used it to analyze all 12 transcripts. After coding transcripts, all authors reviewed coded excerpts to identify patterns and themes throughout interviews. After conducting 12 interviews and observing no new ideas, researchers ceased further recruitment upon achieving thematic saturation with a diverse and nuanced data set.

## RESULTS

### Demographics

We interviewed 12 academic primary care clinicians (10 physicians and 2 NPs) from Internal Medicine (*n*=9) and Family Medicine (*n*=3). Most participants identified as women (*n*=11), had practiced for over 5 years (*n*=10), and all had experienced at least 1 patient death in the past year (Table 1). Interviews were 32 minutes, on average (range 25–38, SD 4.5).

**Table 1:** Characteristics of Study Participants (*n* = 12)

Measure	Item	# Participants
Gender (self-identified)	Women Men	11 1
Years in practice	<5 5–15 15–25 >25	2 4 4 2
Race/ethnicity	White Latinx Black Asian	8 5 1 2
Number of patient deaths in past year	<4 4–10 >10	6 5 1
Degree	MD NP	10 2
Specialty	Internal Medicine Family Medicine	9 3
Patient panel size	200–500 500–1000 >1000	6 5 1
Full-time equivalent (FTE)	100% 80%	10 2

## LOGISTICAL PROCESSING OF PATIENT DEATH IN PRIMARY CARE

### Patient Death Notification Is Inconsistent in Both Timeframe and Method

All participants described inconsistent and varied timeframes with patient death notification. Depending on where patients passed, they were notified hours, days, weeks, or sometimes never after a death. For patients admitted to an associated institution, participants were often able to track patient updates through the electronic medical record (EMR), offer real-time guidance to inpatient practitioners, and knew immediately when a patient died. However, for patients who died at an outside hospital, at home, or in a less expected manner (such as a trauma or suicide), participants often learned of the death weeks to months later, frequently in a jarring and unexpected manner. One participant expressed frustration at the lack of communication from outside institutions stating “*almost never do I get information from an outside hospital…to let me know about a death. Even if they can see I’m the primary care [doctor], even if my patients identify me as their [PCC], it almost never happens*” (participant 11).

In addition to unpredictable timeframes, the way participants were notified also varied. Notification methods included EMR notes, patient family members, hospice workers, clinic staff, medical examiners, and death certificate paperwork in clinic boxes. Participants found it particularly upsetting to unexpectedly learn of a patient’s death from a family member weeks to months after the event. As one participant described “*some people I only hear about [their death] when I call to find out why they didn’t make their… appointment… and when you talk to their family it’s like ‘oh my goodness, I’m so sorry I didn’t know.’ I feel terrible I hadn’t connected with their family*” (participant 7).

Most participants voiced a strong desire that timeframe and notification methods be more efficient, standardized, and humane. One participant lamented “*the thing that I hate is when I come to clinic and get that flash on Epic, saying, ‘patient is deceased.’ I just think…. nobody can let me know, in a way that’s a little bit nicer than a flag on Epic?*” (participant 8).

### There Are a Wide Variety of Logistical Steps Taken After Patient Death Notification

Participants were unaware of post-mortem clinic protocols and reported various logistical practices upon notification including documenting death details, communicating with family, updating staff to mark the patient as deceased, and retrospectively reviewing clinical decisions. One participant noted, “*I try to figure out how, why, and what happened. Both to…just figure it out and to make sure I did not do something horribly wrong in their medical management*” (participant 12).

All participants completed death certificates for patients who died outside healthcare facilities. Many expressed frustrations with extrapolating cause for an unwitnessed death and difficulty navigating complex legal documentation. As one participant stated “*I still forget exactly what you’re allowed to say or not, especially for patients who don’t die in the hospital... And maybe you’re covering for someone, and you don’t know them. Or maybe they are your patient, but you don’t know what exactly caused the death. I always have to Google a refresher of what you can actually put or … [ask] the medical director… what should I actually put for this?*” (participant 9).

Participants felt some responsibility to communicate patient deaths to others. They notified their team’s licensed vocational nurse (LVN), who often knew the patient best other than the PCC, and particularly involved specialists. Almost all participants contacted patient families to offer condolences through cards or calls, and occasionally attended funerals or memorials.

## EMOTIONAL PROCESSING OF PATIENT DEATH IN PRIMARY CARE

### There Are Wide-Ranging Emotional Reactions to Patient Deaths

Participants reported varied emotions upon a patient’s death (summarized in Table 2). Key themes included guilt, rooted in the belief that they could have done more for their patients, and sadness. While a couple participants reported work concentration difficulties, most described intentional emotional detachment to preserve work performance and defer grief processing.

**Table 2:** Emotions Identified by PCCs After Learning of a Patient’s Death

Emotion	Context	Example
Sadness	Especially for long-term patients and families	“*I definitely experienced sadness, and a sense of loss… I felt…sadness for her husband and family as well… remorse, regret… vicarious grief perhaps*” (participant 7)
Relief	When a death was particularly drawn out or painful	“*for patients who have a malignancy or… chronic illness and have been suffering for a long time, there’s always some relief... Like this person was suffering, and hopefully, they had a good death*” (participant 5)
Gratitude	For being part of a patient’s life	“*[A] sense of gratitude for getting to know the patient and their families… I always feel good about the support… I’ve offered to them*” (participant 3)
Numbness	Especially when feeling overworked or burnt out	“*I have an…auto shutdown emotionally… I don’t spend time I probably should. I… shutdown and keep going. A bit of self-preservation*” (participant 2)
Anger	On behalf of patients if PCC felt the healthcare system had failed them	“*I felt...the patient’s death should have been preventable… this patient has suffered at the hands of our system and…‘I can do better’…I felt a lot of anger around that*” (participant 3)
Distress	When how a patient died was not in line with their care goals	“*And they were coded, which was not in alignment with their wishes… That has trauma to it… a feeling of failure on my part… [and] disappointment that they did not have their end of life match their wishes*” (participant 1)
Guilt	Especially when the death was unexpected, or the PCC felt they could have done more	“*For the sudden deaths… they’ve been shocking... For some I felt ashamed because I would doubt myself and feel: ‘Did I do all the things that I could have? Did I miss something? What could have happened that was different?... Why? Why did she die?’*” (participant 8)

Participants recognized various patient and context factors that made deaths more challenging including preventable illness, youth, social vulnerability, blurred doctor-friend roles, and self-identification with patients. Identifying with a recently deceased patient, one clinician noted “*I think part of it is…cultural…I could see my grandfather in him. And I could see similar decisions, hard things that happened with my family around my grandfather’s passing that probably magnified those emotions*” (participant 1). Causes of deaths from COVID, painful conditions, suicides, and overdoses were particularly distressing. As one participant stated, “*I had a younger transgender patient who died from suicide… it was one of those things that sticks with you… I have a lot of responsibility around caring for these patients. And when I don’t do my job… it can be deadly for them*” (participant 10).

### There Is a Need to Find Closure and Connection to Overcome Isolation After a Patient Death

While some participants needed to quickly compartmentalize and move on after a patient’s death, most sought closure, emphasizing the importance of acknowledging the loss and debriefing, given their meaningful relationships with patients. One participant noted “*it’s always a bit weird, because... you’ve had relationships with these patients for… 15-20 years… Then, all of a sudden, there’s none. That feels… very abrupt…Having some sense of closure is super important… Being able to connect with the family, connecting with my team, having [a]… way to mark and memorialize the patient’s passing is important*” (participant 5).

Participants felt isolated in their grief, finding it challenging to create space to emotionally process a patient’s death. One participant reflected “*The weirdest part about the primary care setting is how isolating it is… how alone, you are, unless you create your own way of processing with others. It’s easy to…. be like, ‘Oh, well, that happened, and nobody else knows about it’*” (participant 3). They highlighted the importance of debriefing with others in and outside work, yet expressed concern about burdening non-medical support networks. One participant lamented “*if you’re not in the medical field, it’s…not the same... You don’t want to be the Debbie downer, like, ‘hey, let’s talk about these sad things’*” (participant 6). Participants found debriefing with colleagues and discussing cases with superiors helpful for processing loss but were frustrated by the lack of allocated time for these activities. One participant stated, “*Outpatient isn’t structured in a way where when you have a minute, you can take a pause as a team and debrief*” (participant 1).

Participants were not aware of structured clinical support for processing patient death, and instead created personal coping strategies like connecting with patients’ loved ones, honoring patients through journaling or self-made office memorials, and debriefing with partners, friends, and coworkers. Reflecting on processing patient death, a participant stated, “*When I hear that they died, I sit quietly with my eyes closed, and think about them… saying goodbye to them. Then I make myself a [reminder] about them…. I put in something like ‘this guy loved going to Cache Creek to the casinos’… I give myself…time to grieve for them*” (participant 8).

## CLINICAL SUPPORT RESOURCES

### Need for Structured Clinical Support Resources to Improve Logistical and Emotional Processing

Participants expressed a need for more support resources to process patient deaths logistically and emotionally. One participant noted “*just having structure around [patient death] is helpful, because sometimes what we’re left with is …a ‘whoa, what next’ kind of feeling and it’s nice to be like, ‘Okay, I know what I’m going to do’… Having actionable steps helps me move forward…*” (participant 10). Table 3 outlines suggested resources for logistical and emotional support for both patients and practitioners, and ways to commemorate deceased patients.

**Table 3:** Participant Suggestions for Clinical Support Resources

Logistical resources * Protocolized steps outlining what to do after patient death notification* ○ Prompts for who to notify ○ Instructions for death certificate best practices** ○ Point person to contact if there are questions or concerns following a death * Made available in an efficient and easy-to-access manner* ○ EMR “smartphrases”* ○ Living document in clinic shared folder ○ Case-based onboarding for new PCCs ○ Brief video or document reviewing death certificate “how-tos” ○ Noon conferences with experienced PCCs sharing “best practices”
Emotional support resources * Built-in support for patient families, and venues for honoring deceased patients* ○ Grief packets for patient families ○ Sympathy cards for PCCs to send in real-time ○ Suggestions to support patient loved ones, e.g., attend funeral, donate to charity ○ Organized venues to commemorate patients, e.g., clinic memorial wall, dedicated time during clinic retreats, monthly newsletter blurb, day of remembrance** ○ Built-in considerations for trainee patient deaths, e.g., acknowledgements during team meetings, post-mortem check-ins with clinic attendings * Built-in support for clinicians* ○ On-call support person to debrief about patient loss in real-time** ○ Monthly clinic support groups ○ Automatically shared support resources after each patient death

*Electronic medical record dot phrase that could be typed into a patient chart in real-time with useful information including death documentation template, notification recommendations, resources for patient families, and provider support resource links

**Multiple participants voiced the same recommendation

Participants sought immediate, accessible post-death resources to aid clinicians and patients, suggesting distribution after each death for better utilization, especially after tough losses. They also requested more ways to honor and remember deceased patients. One participant stated, “*It would be.. meaningful… for us to come together and have some remembrance of the patients who passed away… [it] could be sharing a story or a memory… Having some collective coming together and acknowledgement*” (participant 5).

## DISCUSSION

Our findings highlight the challenges PCCs face navigating the logistical and emotional aftermath of ambulatory patient death. Unlike prior research on inpatient death coping strategies, ^3,8,11,12,14^ to our knowledge, this is the first to explore PCC actions post-death notification and recommended resources for comprehensive processing in the primary care setting.

Our research supports prior findings of inconsistent and delayed notification of patient deaths to PCCs, ^21^ dissatisfaction with notification methods, ^21^ and confusion regarding post-death logistics ^25^ and death certificate completion. ^26^ Notably, it contributes to the field by identifying actionable recommendations to improve ambulatory death logistics, including standardizing notifications and providing PCCs with clear post-mortem protocols and educational resources. Despite challenges in matching the immediacy of inpatient death notifications, due to diverse death circumstances outside hospitals, improvements in electronic health record (EHR) interoperability, encouraged by policies like the CURES Act, ^27^ could facilitate easier identification of a patient’s PCC. Further examination of current inpatient-to-PCC patient death notification practices and opportunities for improving timeframe and notice methods is recommended.

Another suggestion was for enhanced logistical support after a patient death. Like clinic protocols for opioid prescribing ^28^ and reducing no-shows, ^29^ guidelines could be made for ambulatory patient deaths. Though perhaps less common than opioid prescriptions or no-shows, the death of a long-term patient can cause significant distress. ^2,7,10,20^ Our research reveals that the current lack of standardized post-mortem guidelines results in idiosyncratic handling of logistical and emotional aspects of patient deaths. Lack of built-in support resources and clear protocols may exacerbate inefficiency, stress in an already difficult situation, and feelings of isolation and burnout. ^30,31^ As prior work demonstrates, chaotic work pace, time pressure, and unfavorable organizational culture can contribute to low physician satisfaction, high stress, and increased burn out. ^31^ Implementing easily accessible post-mortem protocols, such as EHR prompts for notification procedures, death certificate guidance, or clinic staff-sent grief packets, could provide busy PCCs with systematic guidance, saving time and reducing distress in these less frequent situations.

Our study underscores the profound emotional toll of patient death on PCCs, and opportunities to enhance well-being support in primary care, a sector often underemphasized relative to inpatient settings. ^7,16,17,32^ PCCs often forge long-term relationships with patients and their families, manage patients autonomously, and lack a support network familiar with their patients to help navigate loss in real-time. ^7,17,24,25^ Longer patient relationships, while generally more satisfying, with increased closeness, can also heighten emotional impact upon a patient’s death. ^3^ Both inpatient and outpatient clinicians experience grief after a patient’s death, but the enduring relationships of PCCs with their patients likely amplify their risk of prolonged emotional distress. Our study identifies actionable support strategies for PCCs facing challenging losses, including real-time debriefing, an on-call peer, support groups, clinic memorials, and readily available support resources post-death. Recommendations like clinic memorial trees and monthly support groups are especially practical, making them good candidates for initial interventions.

Our findings reveal a profound sense of isolation among PCCs when navigating patient deaths, a sentiment particularly prevalent among outpatient practitioners compared to inpatient peers. ^1,11,12,33^ Although PCCs can of course benefit from peer support, our study highlights the isolation PCCs feel, likely attributable to outpatient care’s distinct dynamics. While inpatient clinicians can benefit from collective, immediate support from an interdisciplinary team well-acquainted with the patient—such as bedside nurses, in-house consultants, and medicine team members ^1^—PCCs typically work one-on-one with patients, without the benefit of a professional network aware of their patient’s situation or passing. Consequently, PCCs must take additional steps to proactively inform their colleagues about their loss to solicit support. Prior studies have also shown that female PCCs are more likely to experience isolation, ^33^ a finding consistent with our predominantly female cohort. Our findings underscore the importance of enhancing social support for PCCs who typically engage in one-on-one patient care. Prior research suggests that healthcare professionals benefit from working in interdisciplinary teams, experiencing higher job satisfaction and reduced burnout risk. ^34,35^ This benefit is particularly apparent among palliative care clinicians who frequently work in an interdisciplinary or even transdisciplinary team format. ^36,37^ While primary care is increasingly moving towards a team-based care model, many primary care teams work in a multidisciplinary—rather than inter- or transdisciplinary—manner, which may result in less collaboration and cohesion. ^38^ Future studies on the role of interdisciplinary teams in managing outpatient clinicians’ grief after patient loss could inform policies to enhance team-based primary care.

Our findings must be interpreted in the context of study limitations. All participants were from clinics affiliated with the same institution in an urban setting and thus represent perspectives shaped by a particular context and culture. Most of our participants are also academic clinicians, meaning they are not 100% clinical and have a variety of research, administrative, and educational roles. Their patient panels and clinic time are therefore considerably lower than the average primary care clinician, and they therefore likely experience fewer annual patient deaths. As with any retrospective interview study, there is also risk that participants inaccurately recalled the events surrounding prior patient deaths.

This study enriches the literature by illuminating the underexplored topic of PCCs’ emotional and logistical processing of patient deaths. Through qualitative analysis, it establishes a foundation for future quantitative research and offers concrete recommendations to enhance support for PCCs experiencing outpatient deaths. Given primary care’s unique traits—such as lasting patient bonds, greater isolation in ambulatory compared to inpatient settings, and increasing burnout rates—monitoring PCCs for burnout and grief related to patient death and enhancing guidance and support for PCCs is vital to reduce administrative strains and grief from patient loss. A dedicated focus on these areas could improve the professional experience of primary care clinicians, aiding them in effectively navigating these challenging occurrences and potentially boosting their resilience in the face of their patients’ mortality.

### Supplementary Information:

Below is the link to the electronic supplementary material.

Supplementary file1 (DOCX 28 KB)

## Data Availability

The data that support the findings of this study are available from the corresponding author upon reasonable request
