# Supplementary material for: How Primary Care Clinicians Process Patient Death: Logistics, Emotions, and Opportunities for Structural Support
Source: J Gen Intern Med. 2024 Mar 8;39(12):2277–83. doi: 10.1007/s11606-024-08702-0 (PMC11347538; doi:10.1007/s11606-024-08702-0)
Supplement: Supplementary file 1 — Supplementary file1 (DOCX 28 KB) [file 11606_2024_8702_MOESM1_ESM.docx]

**APPENDIX**

**PRIMARY CARE CLINICIAN INTERVIEW PROTOCOL**

**Intro and Consent** *(to be read by interviewer at the beginning of each interview)*

Thank you for your willingness to participate in this UCSF study. The purpose of this qualitative research project is to explore primary care clinician’s experiences with patient deaths and to identify resources and support that might benefit PCCs when dealing with patient death.

This interview should last 30-45 minutes and will be audio-recorded and transcribed. Your participation is confidential and all identifying information will be removed from interview transcripts. There are no foreseeable risks to taking part in this study. Potential benefits from participation may include future educational and clinical support resources. You can stop participation in the study at any point throughout the interview.

Do you have any questions before we begin?

May I have your verbal consent to participate in this audio-recorded interview?

**PART 1: Experiences with Death in Primary Care: Logistics**

***I am going to start off by asking you a few questions related to the logistics surrounding patient death in primary care.***

- Can you think of a patient on your panel who died in the last year or two and tell me what happened?
  - How did you find out about their death?
  - How long after their death did you find out?
  - *Typically, in what manner and how long after a patient’s death are you notified?*
- What did you do immediately after finding out that your primary care patient had died?
  - *Can provide prompts:*
    - Contact family? Deep dive in chart? Complete death certification paperwork? Write a note acknowledging receipt of death notice? Notify other clinicians who have cared for the patient?
- Who, if anyone, did you notify of the patient's death?
- How typical was this experience compared to other times when a patient on your panel has died?
- Are there any clinic expectations that you are aware of, in terms of documentation or protocolized steps to take, after a primary care patient dies?
  - Do you think there should be and if so, what might those look like?

**PART 2: Experiences with Death in Primary Care: Emotional Processing**

***Next, I am going to ask a few questions related to the emotional processing surrounding patient death in primary care.***

- Reflecting on the last 2-3 of your patients who died, could you share with me some of the feelings you had when you learned of their deaths?
- Are there any deaths from your primary care panel that were particularly challenging or upsetting?
  - What made these patient losses more difficult than others?
- What strategies have you used to process your feelings after learning of a primary care patient's death?
  - Do you have any rituals or clinical practices you apply routinely to process a patient loss?
- Who, if anyone, have you found it helpful to process your feelings with after a patient loss?
  - *In a professional setting:* Other clinic attendings? Specialists? RNs?
  - *In a personal setting:* Family? Friends? Partners?
- Has the way in which you process a patient’s death changed over time?
  - Do you do anything differently now compared to early on in your practice?

**PART 3: Experiences with Death in Primary Care: Suggestions for Educational Resources and Structured Clinical Support**

***Next, I am going to ask a few questions related to resources that might be helpful for PCCs dealing with patient death.***

- Have you ever received any formalized education or training on how to deal with the death of a primary care patient?
  - Did you receive any training on the logistical steps related to losing a primary care patient?
    - *Prompt: like how you would be notified, if you should chart anything, etc*
  - Did you receive any training related to emotional processing strategies or clinical support resources available after losing a primary care patient?
    - *Prompt: like clinician support groups? Therapist? Day of Remembrance?*
- What type of education or training do you think would be useful to better prepare PCCs for dealing with the **logistics** of losing a primary care patient?
- What type of education or training do you think would be useful to better prepare PCCs for dealing with the **emotional processing** of losing a primary care patient?
- What types of built-in support resources do you think would be helpful to better support PCCs dealing with the loss of a patient in primary care?
  - *Prompts: Clinician support groups? Peer-to-peer organized support? Wellness activities? Remembrance days?*

**PART 4: General Demographic Information**

***We are going to wrap up with a few quick logistical questions.***

- How many years have you been practicing as a PCC?
  - *Ranges: <5, 5-10, 10-15, 15-20, 20-25, 25+*
- What, if any, gender do you identify as?
- What, if any, race, and ethnicity do you identify as?
- How many of your primary care patients have died within the past year?
- *If not obvious from professional profile:*
  - What is your degree (MD, NP, DO)?
  - Specialty (IM vs FM)?
  - Clinical site (DGIM, Lakeshore, etc)
- Do you currently work full or part time?
- Roughly, what is the size of your primary care patient panel?

**PART 5: Wrap up**

Is there anything else you would like to share about this topic?

Do you have any final questions for me?

***Thank you very much for your time, thoughtful responses, and participation!***
